# Complete genome sequences of *Streptomyces griseus* phages Spelly and Phredrick

**DOI:** 10.1128/mra.01049-23

**Published:** 2023-12-19

**Authors:** Joyce Stamm, Julie A. Merkle, Ava Abernathy, Elizabeth Ackerman, John Brown, Abbey Harris, Kayli Hoffman, Ashleigh Hoskins, Abbie Jahn, Nathan Jones, Ashley Kitch, Nandini Mathavan, Nat Rose, Joey Taylor

**Affiliations:** 1 Department of Biology, University of Evansville, Evansville, Indiana, USA; Portland State University, Portland, Oregon, USA

**Keywords:** bacteriophages, *Streptomyces griseus*, soil, Indiana, whole genome sequencing

## Abstract

We present the complete genome sequences of two viruses with siphovirus morphology, isolated from soils collected in Southwestern Indiana using the host *Streptomyces griseus*. Spelly is a BE2 cluster phage with a 131,347-bp genome. Phredrick is a BK1 cluster phage with a 128,873-bp genome.

## ANNOUNCEMENT

Custom phage cocktails have recently shown promise in combating antibiotic-resistant bacterial infections (1, 2). It is, therefore, important to expand the bacteriophage arsenal for various bacteria to provide resources for creating effective bacteriophage treatments. Here, we report on two novel phages that infect *Streptomyces griseus*: Spelly and Phredrick.

**Fig 1:**
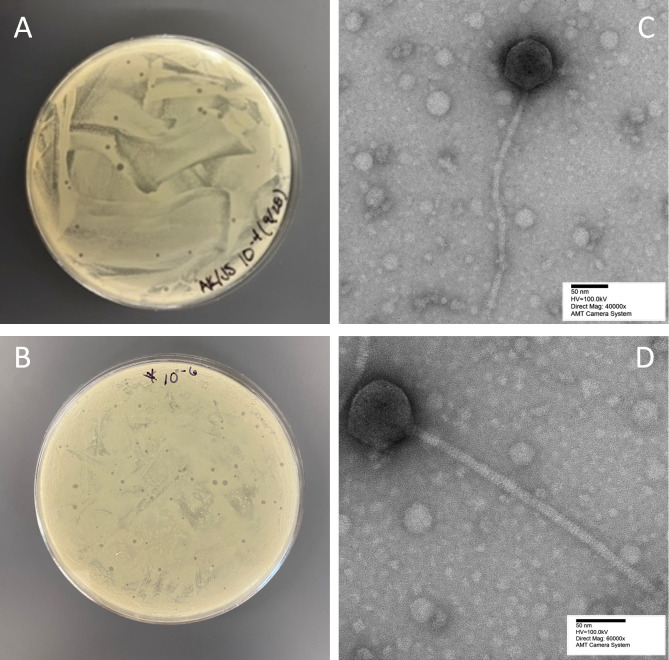
Plaques formed by Spelly (**A**) and Phredrick (**B**) on plates spread with *Streptomyces griseus*. Spelly produces medium-sized plaques, whereas Phredrick consistently produces a combination of small and medium-sized plaques. Transmission electron micrographs of Spelly (**C**) and Phredrick (**D**) stained with 1% uranyl acetate and viewed at 100 kV accelerating potential in a JEOL 1400Plus transmission electron microscope (Western Kentucky University Southern Kentucky Center for Advanced Microscopy). Phage dimensions are provided in Table 1.

Spelly and Phredrick were extracted from dry soil samples collected in Southwestern Indiana in September 2022 (Table 1). Enriched isolation was performed using protocols described in the Phage Discovery Guide (3). Briefly, soil samples were suspended in ~25 mL of nutrient broth supplemented with glucose, MgCl_2_, and Ca(NO_3_)_2_, filtered through a 0.22-µm filter, seeded with 150 μL *Streptomyces griseus* (ATCC 10137), and incubated for 72 hours at 20°C. The enriched culture was plated with host bacteria onto supplemented nutrient agar, and incubated for 2 days at 30°C. Two subsequent rounds of plating were performed to purify each phage, resulting in consistent plaque morphologies. Both Spelly (Fig. 1A) and Phredrick (Fig. 1B) produced clear plaques. Negative-stain transmission electron microscopy using uranyl acetate (1%) indicates that both phages exhibit a siphovirus morphology, with icosahedral capsids and noncontractile tails (Fig. 1C and D; Table 1).

**TABLE 1:** Properties of phages Spelly and Phredrick

Phage	Spelly	Phredrick
Location found	Jasper, Indiana	Chandler, Indiana
Location coordinates	38.44025 N, 87.01399 W	38.0230 N, 87.225.02 W
Capsid diameter [nm ± SD (*n*)]	72.2 ± 5.1 (15)	82.0 ± 3.2 (10)
Tail length [nm ± SD (*n*)]	333.9 ± 16.2 (15)	336.0 ± 22.5 (10)
Genome size (bp)	131,347	128,873
Approximate coverage (×)	789	770
Number of reads	651,700	686,993
GC content (%)	49.4	47.1
Direct terminal repeat length	12,543	765
Cluster	BE2	BK1
Number of protein-coding genes	248	238
Number of tRNAs	44 tRNAs and 1 tmRNA	35 tRNAs and 1 tmRNA
GenBank accession no.	OR725493	OR553903
SRA accession no.	SRX19690860	SRX19690851
Isolated by	K. Hoffman and E. Ackerman	A. Kitch, J. Smith, N. Rose, and A. Harris

Genomic DNA was extracted from high titer lysates using the Promega Wizard DNA cleanup kit and sequenced using the Illumina MiSeq sequencer (v3 reagents) after library preparation with the NEBNext Ultra II FS Kit. Raw 150-bp single-end reads were assembled using Newbler v2.9 (4). Genome completeness and termini were determined with Consed v29 (5, 6). Sequencing and genome details are provided in Table 1. Based on gene content similarity of >35% to phages in the Actinobacteriophage database (https://phagesDB.org/), Spelly was placed in the BE2 subcluster and Phredrick in the BK1 subcluster (7, 8).

The genomes were annotated using DNAMaster v5.23.6 (http://cobamide2.bio.pitt.edu/) embedded with Glimmer v3.02b (9) and Genemark v2.5 (10), PECAAN v1.0 (discover.kbrinsgd.org), Phamerator database version 505 (11), Starterator database version 505 (http://phages.wustl.edu/starterator/), BLASTp (Actinobateriophage, NCBI non-redundant databases) (12), HHPred (PDB mmCIF70, SCOPe70, Pfam-A, NCBI Conserved Domain databases) (13), TMHMM (14), and SOSUI (15). Aragorn v1.2.41 (16) and tRNAscanSE v2.0 (17) were used to identify tRNAs. Default parameters were used for all software.

Spelly has 248 predicted protein-coding genes, of which 28% could be assigned putative functions. Similar to other BE cluster phages, Spelly has a long terminal repeat, and the first 27 genes in the genome are identical in sequence and position to the last 27 genes. Spelly contains 44 predicted tRNAs, representing all 20 naturally occurring amino acids, and 1 tmRNA.

Phredrick has 238 predicted protein-coding genes, of which 35% could be assigned putative functions. Similar to other BK1 phages, Phredrick contains a predicted programmed (−1) translational frameshift that produces two tail assembly chaperone proteins that share a start codon. Phredrick contains 35 predicted tRNAs, representing all 20 naturally occurring amino acids, and 1 tmRNA.

Consistent with BE2 and BK1 cluster phages, no integrase or immunity repressor functions were identified in either genome. This, together with the clear plaque morphologies observed, suggests that both phages have lytic life cycles.

## Data Availability

Spelly is available at GenBank with Accession No. OR725493 and Sequence Read Archive (SRA) No. SRX19690860. Phredrick is available at GenBank with Accession No. OR553903 and Sequence Read Archive (SRA) No. SRX19690851.
